# Institutional factors affecting participation in national faculty development programs: a nation-wide investigation of medical schools

**DOI:** 10.1186/s12909-017-0888-1

**Published:** 2017-02-28

**Authors:** Do-Hwan Kim, Jinyoung Hwang, Seunghee Lee, Jwa-Seop Shin

**Affiliations:** 1 Department of Medical Education, Seoul National University College of Medicine, 103 Daehak-ro, Jongno-gu, Seoul 03080 Republic of Korea; 2 Department of Educational Psychology, The University of Texas at Austin, 1 University Station, MS/D5800, Austin, TX 78712 USA

**Keywords:** Faculty development program, Educational goals, National Teacher Training Center, Faculty members

## Abstract

**Background:**

Medical schools have used faculty development programs as an essential means to improve the instruction of faculty members. Thus far, however, participating in such programs has been largely voluntary for individuals even though a certain degree of participation is required to achieve practical effectiveness. In addition, the learning behaviors of faculty members are known to be influenced by organizational contexts such as a hidden curriculum. Therefore, this study explored the organizational characteristics of medical schools affecting attendance at faculty development programs.

**Methods:**

Forty medical schools in South Korea were included in this study. In total, 1,667 faculty members attended the faculty development programs at the National Teacher Training Center for Health Personnel between 2007 and 2015. For independent variables, information on the basic characteristics and the educational states was collected from all the medical schools. Themes were identified from their educational goals and objectives by inductive content analysis.

**Results:**

The number of nine-year cumulative attendees from medical schools ranged from 8 to 104. The basic characteristics of the medical schools had little influence on faculty development program attendance, while several themes in the educational goals and objectives, including “cooperation”, “serving various societies”, and “dealing with a changing future” showed a significant difference in participation. The number of full-time faculty showed a significant positive correlation when it was smaller than the median, and the proportion of alumni faculty showed a significant negative correlation when it was higher than 50%.

**Conclusions:**

This study adds to existing knowledge on factors affecting attendance at faculty development programs by identifying related institutional factors that influence attendance. While the variations depending on the basic characteristics were minimal, the organizational environment surrounding medical education significantly contributed to attendance. Addressing institutional as well as individual factors could contribute to improving participation by faculty members in faculty development programs.

## Background

Medical schools have used faculty development programs (FDPs) as an essential means to manage and assist their faculty members to adapt to changes in internal and external environments [1, 2]. While researchers have defined FDPs in a variety of ways, instructional development has steadily been the central focus of faculty development [3–5], and in practice, FDPs that prepare faculty members as medical teachers have been acknowledged as “imperative” for all medical schools [5].

However, the simple existence of FDPs does not ensure educational improvement by itself. In order for a FDP to facilitate teaching improvement at an organization, a certain degree of participation should be ensured [6]. From the perspective of individual faculty members, this is because attending FDPs is like “grammar school for teachers” to use a metaphor and an efficient way of obtaining at least the minimum required knowledge and skill needed to function as a medical teacher [6]. Second, for a medical school, having a critical mass of educators is vital because it helps to promote changes in the educational environment [7]. For instance, it has been argued that cumulative FDP attendees contribute by supporting critical periods such as curricular reform by forming a community of educators [8]. Third, for faculty developers, securing a certain level of participation is a practical issue because the operation of FDPs involves fixed expenses regardless of the number of paid attendees. Given that limited financial resources is one of the most common challenges faced by faculty developers [9], recruiting participants and charging registration fees for individuals or their affiliated organizations may help offset the expenses and mitigate the financial burden on faculty developers [10, 11].

Despite the importance of participating in FDPs, one problem regarding FDP attendance is that frequently the decision to attend is a voluntary one made by the individual rather than based on deficiencies in teaching skills or the need for improvement in the individual, which results in the case where “those who need faculty development the most attend the least” [12]. This case could be similar at the organizational level. It was reported that the manner in which institutions use FDPs is a manifestation of the institutions’ inner faith in their workforce [2]. However, faith may not always correspond with their objective needs. More importantly, given that the learning behaviors of faculty members are affected by hidden curriculum comprised of various informal-tacit elements [13], the educational environment may exert a stronger influence than that of individual needs or interests in participating in FDPs.

For these reasons, it was suggested that studies on FDP participation should move beyond individual factors which basically rely upon volunteerism and delve into the “social determinants of participation” [14]. The significance of organizational context in faculty development was also underlined in a more recent article by O’Sullivan & Irby, in which the authors called for process-oriented studies encompassing faculty development and workplace communities [15].

To the best of our knowledge, however, studies on FDP attendance are not only scarce but have also only focused on identifying factors mainly at the individual level [6, 16, 17]. One of the reasons for this could be in the case for which the majority of FDPs are institutional [18] where organizational factors of participation are difficult to isolate from individual factors due to their ubiquitous influence on participants. On the other hand, in South Korea, due to the existence of the National Teacher Training Center for Health Personnel (NTTC), the only representative institution specialized for faculty development, FDPs for medical schools have been done mainly at the national level which is relatively suitable for identifying institutional influences on participation. Since its inception, the NTTC has been provided with not only solid support from the domestic medical education community but also with external assistance from the WHO West Pacific Regional Office in 1970s and 1980s to strengthen the institution [19]. Furthermore, the unique geographical context of South Korea, where the area is the smallest among the top twenty countries with the highest number of medical schools [20], also has contributed to minimize the barrier to participation which originates from geographical distance.

Nonetheless, because medical schools generally do not have any mandatory requirement for NTTC FDP participation, the choice to attend FDPs was almost entirely decided by the medical schools and their members. Consequently, it resulted in considerable variation in the number of attendees among medical schools. Given this situation, the aim of this study was to examine the relationship between the attendance of NTTC FDPs by organizations and their characteristics, specifically for three types of factors – basic characteristics, educational goals and objectives, and educational states.

## Methods

### Setting

The NTTC, founded in 1975, is the leading institution for faculty development in South Korea. When it was first decided to set-up the NTTC at the Deans meeting for Korean Medical Schools, the primary purpose was to establish an institution specializing in the education and training of health professionals [21]. Based on the initial purpose of the NTTC, its programs have consistently been available to external domestic institutions despite the NTTC’s status as an annex of Seoul National University College of Medicine (SNUCM). As the institution grew, a standard pattern for the NTTC FDPs was gradually established which can be characterized as (1) a workshop or a series of workshops (2) that are conducted at least once or twice a month (3) and are usually one or two days long for each program.

### Sample

Participants in the NTTC FDPs between 2007 and 2015 were examined in this study. During this period, 4,195 faculty members participated in 158 workshops. Among them, we excluded 2,134 SNUCM faculty members because their participation was often compelled by an institutional promotion criteria or educational policy due to the exceptional relationship between the NTTC and SNUCM unlike the other medical schools. From the remaining 2,061 attendees, 394 faculty members who were from non-medical schools such as a school of dentistry or a school of nursing were also excluded. Finally, the collection and analysis of data were done for 1,667 faculty members from 40 medical schools in South Korea.

### Characteristics of medical schools

#### Basic characteristics

We chose to include fixed or relatively stable features of a medical school for the basic characteristics. In this category, we collected the following information – ownership (national/public vs private), location (capital area vs non-capital area), adoption of a graduate-entry program (GEP) (adopted vs not adopted), and founding year of the institution. When collecting the data, we referred to the relevant web pages at the official website for each school.

#### Educational goals and objectives

In 2015, three of the authors (DHK, JH, SL) identified 13 themes included in the educational goals and objectives of medical schools in South Korea with inductive content analysis [22]. Inductive content analysis is a qualitative research method used to create a conceptual system or categories which help to obtain a condensed description of textual data. This process is reductive and comprised of open coding, grouping, categorization, and abstraction [23, 24]. In the present study, the authors reviewed and revised the existing results of Kim et al. [22]. The themes were iteratively discussed until the differences were resolved; thus, finally, the medical schools were grouped according to their inclusion in each theme for the educational goals and objectives.

#### Educational states

To determine the independent variables included in this category, we examined and iteratively discussed items in the latest version of *The Current State of Medical Education* (16^th^ edition, exclusive for the Korea Association of Medical Colleges members only) published in 2012 and a white book published by the Research Institute for Healthcare Policy of Korean Medical Association in 2014 [25] as the principal sources of references. Similar to the basic characteristics, features that are essentially unchanging or stably maintained were mainly considered. Moreover, we gave consideration to whether documented information is integral and quantifiable. Finally, the data collected from each medical school included the size of a class, the established year of the department of medical education (DME), the length of the formal curriculum, and the number of full-time faculty. Regarding the faculty members, the proportion of alumni faculty, which was defined as faculty members currently employed by their alma mater medical school, was calculated as well.

### Statistical analysis

In this study, independent variables were various characteristics of medical schools and dependent variable was the number of cumulative attendees from 2007 to 2015. However, contrast with basic characteristics or educational states which are relatively stable, medical schools occasionally review and revise their educational objectives to meet changing internal and external needs. Therefore, additional dependent variable that focuses on more recent data, the number of cumulative attendees from 2013 to 2015, was also analyzed as dependent variable to confirm the consistency of the findings. Lastly, we investigated if the number of participants in year N has correlation with that in year N-1 and cumulative participants from 2007 to year N-1.

Although this study to reach a near nation-wide investigation that embraces all medical schools only except SNUCM, the sample size was inevitably restricted by their total number. Nevertheless, because parametric statistics is highly robust even when the assumptions with regard to sample size or normality are violated [26], independent sample *t*-test or Pearson correlation was used when independent variables were dichotomous or continuous, respectively. *P*-values less than 0.05 were considered statistically significant.

## Results

### Characteristics of medical schools

Since 1998, there have been a total of 41 medical schools including SNUCM in South Korea, and all of the medical schools had at least eight faculty members who participated in the NTTC FDPs between 2007 and 2015 (Table 1). The average number of attendees per medical school was 42.1, and the median was 37. Among the 40 medical schools, nine were national/public; fourteen were located in the capital area, and twenty-six adopted a graduate-entry program. The years for establishment ranged from 1885 to 1998, and the median was 1981. Among the 13 themes that were identified through the inductive content analysis, each medical school had 8.3 themes on average in its educational goals and objectives. Approximately two-thirds of the medical schools had DMEs with a median year of establishment of 2004. The medical schools admitted on average 73.1 students per year and had 260 full-time faculty members, among which 41.4% were alumni faculty.

**Table 1:** Descriptive statistics for 40 medical schools in South Korea

Categories			Total medical schools (100%, *N* = 40)^a^
FDP participants	Number of cumulative participants from 2007 to 2015		42.1 ± 27.7 (range: 8–104)
Basic Characteristics	Ownership	National/Public	22.5% (9)
		Private	77.5% (31)
	Geographical position	Capital area	35.0% (14)
		Non-capital area	65.0% (26)
	Graduate-entry program	Adopted (partly or fully)	65.0% (26)
		Not adopted	35.0% (14)
	Founding year of medical school		1975.8 ± 22.6 (range: 1885–1998)
Educational goals and objectives	Number of themes included (Maximum: 13)		8.3 ± 2.07 (range: 4–13)
Educational states	Size of a class (*n*)		73.1 ± 29.8 (range: 40–125)
	Established year of DME^b^	Not established	n.a
		yet Established	2004 ± 4.53 (range: 1995–2012)
	Length of formal curriculum (weeks)		152.1 ± 12.1 (range: 120–183.6)
	Number of full-time faculty (*n*)		260.0 ± 175.0 (range: 89–863)
	Percentage of full-time faculty who graduated from their current affiliated medical school (i.e., alumni faculty)		41.4 ± 32.2 (range: 0.18–95.0)

*FDP* faculty development program, *DME* department of medical education

^a^Among 41 medical schools in South Korea, Seoul National University College of Medicine, where NTTC is located, was excluded in the statistics; ^b^ This excludes any administrative office related to medical education, such as “Office of Medical Education”. In total, among 40 medical schools, 26 had DME or similar entity

### Influence of basic characteristics

We examined the variation in the 9-year cumulative number of NTTC FDP attendees of medical schools depending on their basic characteristics (Table 2). The numbers of attendees, however, showed no statistically significant difference irrespective of a medical school’s ownership, geographical position, adoption of a graduate-entry program, and founding year.

**Table 2:** The influence of various basic characteristics of medical schools

Variable		Cumulative participants from 2007 to 2015^a^	
		Values	*p*-value
Ownership^a^	National/Public	41.77 ± 27.89	0.884
	Private	43.33 ± 28.39	
Geographical position^a,b^	Capital area	41.38 ± 26.85	0.821
	Non-capital area	43.5 ± 30.06	
Graduate-entry program^a,b^	Adopted (partly or fully)	44.57 ± 30.96	0.687
	Not adopted	40.80 ± 26.24	
Founding year of medical school^c^	Correlation	0.049	0.762

*FDP* faculty development program

^a^ Mean ± SD; ^b^ independent sample *t*-test; ^c^ Pearson correlation

### Influence of themes in the educational goals and objectives

To evaluate the effect of the educational mission, the FDP attendance of medical schools was compared according to whether a certain theme was contained in the educational goals and objectives (Table 3). A statistically significant or near-significant difference in the number of attendees was observed in three of the thirteen themes. First, medical schools that highlighted “cooperation” in education had a higher attendance for both dependent variables compared to those who did not. In contrast, when “serving various societies” or “dealing with a changing future” was included, there were significantly less attendees than when the theme was not included.

**Table 3:** Themes in the educational goals and the number of attendees

			Cumulative participants from 2007 to 2015		Cumulative participants from 2013 to 2015	
			Values	*p*-value	Values	*p*-value
Total number of themes included		Correlation^a^	−0.223	0.167	−0.264	0.100
Themes^b, c^	Medical expertise^d^		n.a.		n.a.	
	Professionalism	Not included (38)	67.66 ± 43.82	0.097	26.33 ± 25.50	0.416
		Included (2)	40.05 ± 25.74		11.35 ± 9.45	
	Serving various societies^e^	Not included (36)	77.75 ± 41.24	0.005	28.0 ± 14.44	0.003
		Included (4)	38.16 ± 23.34		10.75 ± 9.91	
	Self-management and development	Not included (35)	48.5 ± 35.06	0.547	12.5 ± 10.54	0.995
		Included (5)	41 ± 26.61		12.47 ± 11.78	
	Founding philosophy	Not included (28)	53.76 ± 25.60	0.064	15.92 ± 16.37	0.304
		Included (12)	36.51 ± 27.26		10.81 ± 8.067	
	Research ability	Not included (28)	49.92 ± 30.82	0.220	15.15 ± 11.75	0.312
		Included (12)	38.37 ± 25.75		11.18 ± 11.33	
	Cooperation	Not included (26)	31.28 ± 17.37	0.034	8.64 ± 6.70	0.066
		Included (14)	47.96 ± 30.56		14.53 ± 13.02	
	Leadership	Not included (24)	44.37 ± 26.41	0.680	11.31 ± 11.17	0.607
		Included (16)	40.62 ± 28.90		13.25 ± 11.84	
	Dealing with a changing future	Not included (20)	49.71 ± 31.27	0.063	16.28 ± 13.44	0.023
		Included (20)	33.73 ± 20.68		8.26 ± 6.99	
	Respect for life	Not included (20)	40.75 ± 27.73	0.758	14.05 ± 13.40	0.392
		Included (20)	43.5 ± 28.21		10.9 ± 9.244	
	Creativity	Not included (20)	40.95 ± 24.37	0.782	11.09 ± 9.787	0.431
		Included (20)	43.42 ± 31.51		14 ± 13.20	
	Problem-solving ability	Not included (19)	39.33 ± 25.15	0.509	13.90 ± 11.52	0.415
		Included (21)	45.21 ± 30.56		10.89 ± 11.52	
	Ability of education	Not included (6)	43.82 ± 28.54	0.309	13.14 ± 11.97	0.337
		Included (34)	30.2 ± 17.99		7.8 ± 5.890	

*n.a*. not available

^a^Pearson correlation; ^b^independent sample*t*-test; ^c^ Mean ± SD; ^d^This theme was included in educational goal of 39 out of 40 medical schools; ^e^This includes local, regional, national, and international societies

### Influence of educational states

Among factors related to medical education, the number of cumulative attendees was not significantly correlated with either the size of a class or the length of the formal curriculum (Table 4). Whether a medical school has a DME did not show any significant influence; however, when an analysis was conducted with the 26 medical schools that do have a DME, DMEs that were established more recently had more faculty members attending the NTTC FDPs.

**Table 4:** The influence of educational states

Variable			Cumulative participants from 2007 to 2015	
			Values	*p*-value
Size of a class^a^			0.142	0.382
Length of formal curriculum (weeks)^a^			−0.189	0.248
DME	Establishment^b^	Not established yet	38.64 ± 30.32	0.566^c^
		Established	44.0 ± 26.53	
	Established year of DME^a^		0.397	0.045
Number of full-time faculty^a^	Total number of full-time faculty		0.167	0.302
	Total faculty < median		0.460	0.041
	Total faculty ≥ median		0.267	0.256
Proportion of full-time faculty who graduated from their own medical school^a^	Proportion of alumni faculty		0.008	0.961
	Proportion < 50%		0.165	0.476
	Proportion ≥ 50%		−0.670	0.006

*DME* department of medical education

^a^ Pearson correlation with cumulative participants from 2007 to 2015; ^b^ Mean ± SD ^c^ independent sample *t*-test

With respect to faculty members, both the number of full-time faculty and the proportion of alumni faculty did not show any significant correlation with FDP attendance overall. Nevertheless, a different result emerged when we restricted the range of the independent variables. First, the number of full-time faculty showed a significant positive correlation only when the number of full-time faculty was smaller than the median number of full-time faculty, which was 189. Second, the proportion of alumni faculty had a significant negative correlation only when the proportion of alumni faculty was higher than 50%.

### Correlation between the number of attendees

The correlation between the number of N^th^ year attendees and N-1^th^ year attendees was significant with a strength that ranged from 0.360 to 0.808 except for three of the eight cases (Table 5). Likewise, the cumulative total of the N-1^th^ year was also positively correlated with the number of attendees of the N^th^ year except for three cases. In this case, the strength of the correlation ranged from 0.390 to 0.706.

**Table 5:** Correlations between the number of attendees

	Number	2008	2009	2010	2011	2012	2013	2014	2015
N-1^th^ year – N^th^ year^a^	Correlation	0.646	0.808	0.360	0.221	0.216	0.020	0.614	0.509
	*p*-value	0.000	0.000	0.023	0.170	0.181	0.902	0.000	0.001
Cumulative total of N-1^th^ year – N^th^ year^a^	Correlation	0.646	0.706	0.289	0.236	0.178	0.437	0.390	0.509
	*p*-value	0.000	0.000	0.071	0.143	0.273	0.005	0.013	0.001

^a^Pearson correlation

## Discussion

In this study, we investigated the institutional factors affecting participation in national faculty development programs. The results indicate that while the influence of the basic characteristics of medical schools is insignificant, several factors regarding educational missions, including the established year of a DME and the quantitative and qualitative features of faculty members, significantly affected the FDP attendance of medical schools. Moreover, not only the cumulative attendance but also the number of attendees in a specific year predicted the number of attendees for the following year.

The first finding of this study is that four factors, which we categorized as basic characteristics, have little impact on the number of FDP attendees. This might seem unusual because the features of medical schools including ownership [27], geographic position [28], type of M.D. granting program [29], history and tradition [30] are often reflected in the outcome or orientation that institutions pursue by means of education. However, taking into account the history of the growth of medical schools in South Korea over the last few decades, the results are rather reasonable. For instance, the rapid expansion of medical schools, which took place over a relatively short period, has resulted in an immature social agreement on the accountability of medical schools [31]. This, in turn, has brought about the current circumstance for which it is hardly possible to differentiate between national/public and private medical schools in terms of their educational missions [22] as well as their actual curriculum [32]. Another reason could be procedural weaknesses in the implementation of major policies such as establishing new medical schools or introducing GEPs. Researchers have pointed out that there was frequent interference from political and economic interests instead of using evidence-based research on education in the process for adopting those policies [33]. Furthermore, mutual understanding between the government and medical schools with regard to the background and aims of the policies was inadequate also.

However, compared to the basic characteristics, elements that are more closely connected to medical education showed an association with FDP attendance. First, the number of FDP attendees was higher for medical schools that have “cooperation” in their educational goals and objectives. It has been argued that FDPs increase cooperative interactions among faculty members [14]. Moreover, given the large numbers of faculty members involved, integration of curriculums by the joint contribution and harmony of teachers is necessary especially in this era of competency-based medical education [34, 35]. Namely, medical education is a ‘team sport’ whose excellence is more likely to be achieved through cooperative effort for shared values rather than through a mere collection of independent individuals [36]. Therefore, medical schools might utilize FDPs more actively if they appreciate “cooperation” as a primary educational goal.

In contrast, there were fewer FDP attendees when medical schools emphasized “serving various societies” or “dealing with a changing future”. These objectives are more focused on external stakeholders or extrinsic stimuli compared to “cooperation” which is more intrinsic. It is natural for a medical school to properly adjust oneself to the ever-changing needs of the external environment [37] given the imposed expectation to fulfill the social contract with the public [38]. However, considering the limited available resources of faculty members and medical schools [39], the negative association of the extrinsic educational objectives on FDP attendance implies that a delicate balance is required when fulfilling the needs of external stakeholders and developing the internal capacity of faculty members concurrently.

In addition to educational goals and objectives, several features relevant to the educational environment were also correlated with FDP attendance. Unlike the founding year of the medical schools, the year when a DME was established had a significant correlation with the cumulative number of attendees. In other words, FDP attendance was significantly decreased as the chronological age of a DME increased. It could be primarily explained by the organizational capability for running FDPs. Given that one of the major roles of a DME is to educate and train medical teachers [5], a DME with a longer history may have accumulated enough experience and resources so that it could independently perform the role without relying on the NTTC FDPs while a newer DME requires more support. On the other hand, however, it is also worth considering that established traditions often impede achieving meaningful changes in an organization by hindering it from learning new things and reinventing itself [40]. Briefly, the negative effect of the increasing age of a DME exerted on the NTTC FDP attendance could be attributed to either the sufficient capacity of a long-standing DME or its liability in maintaining the status quo which needs further investigation.

Regarding the number of faculty members, it would be intuitive to expect that the number of FDP attendees will keep increasing as the number of faculty members increases, if assuming that only individual factors affect participation. However, our findings show that the relationship was valid only for those medical schools whose number of faculty members was under the median number of faculty members. This implies the possible existence of influences stemming from size-related factors which is consistent with previous studies which indicated that the large size of an organization tends to negatively affect implementing institution-wide behavioral changes [41, 42]. For medical schools, it becomes more complicated due to their three connected missions – education, research, and clinical service – which are interdependent but sometimes conflict with each other [43]. This tension may prevent medical schools from increasing investment in education in order to pursue the two other missions simultaneously. In addition, the fact that the largest school has ten times as many faculty members as that of the smallest one (89 vs 863), which is a far greater difference than the difference in the number of entering students (40 vs 125), might also cause the FDP attendance of medical schools to plateau.

The composition of the faculty members was also important in FDP participation. First, the number of attendees showed a significant negative correlation with the proportion of alumni faculty when it was above 50%. A problem with the so-called “pure-bloodism” could be the homogenization of learning experiences among faculty members because their experiences as learners are the primary factor that shapes their teaching style [44]. In such medical schools, it is easier for junior faculty members to “inherit” the teaching style of senior faculty members through the medium of learning experiences which can become prevalent in organizational practices. In relation to FDP participation, this homogenization is particularly challenging for organizations as well as faculty developers, because it could promote group conformity which often results in faculty members failing to recognize the necessity of improving their current practices, or even worse, remaining silent even when they recognize that corrective action is necessary [45].

Second, our data show that the number of FDP attendees in a certain year significantly predicted the number of FDP attendees in the next year. The exceptions were for the period when the NTTC concentrated its resources on internal capacity building of the SNUCM faculty at the expense of national-wide programs. Of note is that those correlations were repeatedly observed over the years implying that a medical school’s inclination for FDP attendance is a relatively stable trait. Researchers have reported that peer support and a feeling of connectedness with a community of educators are crucial for the development of faculty members as teachers [46]. In this aspect, one of the reasons for a constantly low FDP attendance could be the scattered distribution of the scarce FDP attendees which, in turn, hinders the formation of a community of peers that motivates self-development as a teacher. This is consistent with previous findings that emphasized a critical mass of educators is a key factor in making behavioral changes [7, 47].

### Implications for faculty developers

One of the findings, seemingly obvious yet important, is that there is great variation between medical schools in their NTTC FDP attendance. Moreover, it seems clear that there are medical schools with low attendance, especially showing a continuous trend for low attendance. Systematic reviews, which used the Kirkpatrick model for the evaluation, have already proven that FDPs have substantial beneficial effects over all levels [14, 48]. However, one thing, which should not be overlooked, is that even the level 1 outcome, i.e., satisfaction, is unable to take place unless participation is assumed. Similarly, Marbach-Ad et al. have suggested a five-level model to evaluate teaching and learning programs, which included “participation” on its first level to understand who participates and why individuals participate followed by “satisfaction” on the next level [49]. It is therefore necessary for faculty developers to pay more attention to participation as “level 0” of the evaluation, especially for those with persistent low attendance.

To promote the participation of faculty members, the first thing we need to understand is what encourages them to participate in FDPs. Although researchers have already identified various reasons for participation at the individual level [6, 16, 17], our results show that institutional factors also affect FDP participation. The fact that the number of FDP attendees no longer proportionally increases once the size of the faculty reaches a certain point also implies that institutional factors are operating in addition to individual ones. Therefore, when faculty developers seek more medical teachers to take part in FDPs, addressing personal factors might not be sufficient, and institutional contexts affecting FDP attendance must also be taken into consideration.

Lastly, another recent trend that makes institutional factors more important is the expansion of the scope of FDPs from institutional to national or even international [18]. Similar to individuals having diverse reasons for attending FDPs, each medical school would have different types and combinations of institutional factors, i.e., a hidden curriculum that influences faculty behaviors. Therefore, as Steinert pointed out, faculty developers who want to broaden their audience would need to use targeted marketing strategies depending on their target institutions [50]. For example, when selecting new targets for promoting their programs, medical schools with factors that contribute positively to FDP participation could be considered as a priority for marketing efficiency, such as institutions that have recently established a DME, are not too large, or emphasize “cooperation” in their educational goals. Meanwhile, for existing participating institutions whose trend for attendance is already known, identifying the institutional factors related to education as well as examining the characteristics of previous attendees would be required as baseline data to refine the marketing strategies for a specific institution.

### Limitations

The first limitation of this study is that it was conducted based on data from a single institution, the NTTC, which aims to serve medical schools across South Korea. Thus, the socio-historical context of the institution or nation could have been reflected in the findings. However, the exceptional context consisting of a single national institution specialized for faculty development also offered a favorable setting for a nation-wide study. Second, values collected for some of the independent variables are not permanent because medical schools are constantly changing to adapt to changes in the environment. However, after the implementation of the GEP in 2005, South Korea has maintained a relatively stable medical education environment. Additionally, we used 9-year cumulative attendees as the dependent variable so that the effect due to the year-to-year variation remains minimal. Third, the causal inferences from our correlational relationships could be limited, especially when factors such as organizational capability for running FDPs mediate the relationship. In addition, some institutional factors might influence only specific types of FDPs. Even though it could not be examined due to the difficulties in categorizing NTTC FDPs that often cover multiple topics in a single program, in further studies, it would be worth exploring how the effect of institutional factors on participation varies by the types of programs.

## Conclusions

In this study, we investigated factors at medical schools that influence participation in FDPs. Although medical schools did not show any variations based on their basic characteristics, the organizational environment surrounding medical education, such as the educational missions, the history of a DME, and the qualitative and quantitative features of faculty members, had a significant contribution on FDP attendance. Although the context of the NTTC and South Korea might be a limitation, it seems evident that faculty developers should not ignore institutional factors to promote medical faculty buy-in. Further research is needed to reveal additional institutional factors as well as the mechanisms underlying them.

## Abbreviations

DME: Department of medical education
FDP: Faculty development program
GEP: Graduate-entry program
NTTC: National Teacher Training Center for Health Personnel
SNUCM: Seoul National University College of Medicine
UEP: Undergraduate-entry program
